# Cost-Effectiveness of COVID-19 Sequential Vaccination Strategies in Inactivated Vaccinated Individuals in China

**DOI:** 10.3390/vaccines10101712

**Published:** 2022-10-14

**Authors:** Yaqun Fu, Jingyu Zhao, Xia Wei, Peien Han, Li Yang, Tao Ren, Siyan Zhan, Liming Li

**Affiliations:** 1 School of Public Health, Peking University, Beijing 100191, China; 2 London School of Hygiene & Tropical Medicine, Keppel Street, London WC1E 7HT, UK; 3 Peking University Center for Public Health and Epidemic Preparedness and Response, Beijing 100191, China

**Keywords:** COVID-19, booster, sequential vaccination, cost-effectiveness, Markov model

## Abstract

To effectively prevent and control the COVID-19 pandemic, countries have adopted a booster vaccination strategy. This study aimed to estimate the cost-effectiveness of sequential booster COVID-19 vaccination compared to two-dose inactivated vaccination in China from a societal perspective. A Markov model was developed to estimate the cost-effectiveness of sequential vaccination, including two doses of an inactivated vaccine followed by a booster shot of an inactivated vaccine, adenovirus vectored vaccine, protein subunit vaccine, or mRNA vaccine. The incremental effects of a booster shot with an inactivated vaccine, protein subunit vaccine, adenovirus vectored vaccine, and mRNA vaccine were 0.0075, 0.0110, 0.0208, and 0.0249 QALYs and saved costs of US$163.96, US$261.73, US$583.21, and US$724.49, respectively. Under the Omicron virus pandemic, the sequential vaccination among adults and the elderly (aged 60–69, 70–79, over 80) was consistently cost-saving, and a booster shot of the mRNA vaccine was more cost-saving. The results indicate that the sequential vaccination strategy is cost-effective in addressing the COVID-19 pandemic, and improving vaccination coverage among the elderly is of great importance in avoiding severe cases and deaths.

## 1. Introduction

Coronavirus disease 2019 (COVID-19) is a novel infectious disease caused by severe acute respiratory syndrome coronavirus-2 (SARS-CoV-2), which was announced as a Public Health Emergency of International Concern (PHEIC) on 30 January 2020 and characterized as a pandemic on 11 March 2020 by the World Health Organization (WHO). It is a serious crisis and severe test for the world and has had a huge impact on the global economy. As of 19 August 2022, the WHO reported approximately 591.68 million cumulative cases and 6.44 million cumulative deaths worldwide [1].

A vast majority of countries have provided vaccinations to safeguard the population and economy throughout the pandemic. A regular vaccination strategy was proven to be cost-saving [2,3,4] and cost-effective [5,6] in both high-income countries (HICs) and in low- and middle-income countries (LMICs) [7,8]. However, with the variation in viruses across the globe and the waning vaccine effectiveness (VE) against viruses providing booster vaccination to fully vaccinated populations have been considered in many countries. It was found that the effectiveness of the vaccine decreased over time [9]. Although antibodies can be detected after one year of two-dose vaccination, the level decreased dramatically [10]. Internationally, clinical trials have evaluated the safety and efficacy of a booster dose against different virus strains [11,12,13,14,15,16], and real-world studies have also shown that people with a booster dose have a lower infection rate, hospitalization rate, critical rate, and mortality [17,18,19].

In this study, sequential vaccination refers to a booster dose that differs from an inactivated vaccine, and homogeneous booster vaccination refers to a booster dose of an inactivated vaccine. A booster dose of a heterogeneous type of vaccine was adopted in some countries as a way to address insufficient vaccination supply, which was found to be safer with better immunogenicity, more efficacy, and more flexibility compared with the homogenous booster [20]. This finding was supported by an increasing number of clinical trials and real-world evidence (RWE). In conclusion, sequential vaccination, both with a homogeneous booster [12,14,15,16,19,21,22] and a heterogeneous booster [20,23,24,25,26,27,28,29], was proven to be more effective and safer than the initial inoculations. However, scarce evidence exists about the cost-effectiveness of booster vaccinations, and there are only limited data about the effects among elderly individuals. Most notably, while the Omicron strain of the virus is currently dominating the pandemic, completing the whole population booster vaccination is of greater urgency, especially among vulnerable groups.

China has offered free vaccination to cover the whole population since January 2021. The first step was to cover people aged 18–59, gradually extending to those aged over 60 in April 2021, to adolescents aged 12–17 in July 2021, and to children aged 3–11 in November 2021. Since then, the government has provided booster vaccination since October 2021 and sequential vaccination since February 2022. As the market share of inactivated vaccines (Sinovac CoronaVac and BBIBP-CorV) in China is approximately 90%, this study only explored the cost-effectiveness of a booster shot with an inactivated vaccine, adenovirus vectored vaccine, protein subunit vaccine, or mRNA vaccine among the two-dose inactivated vaccinated population. Except for the mRNA vaccine, all other types of COVID-19 vaccines are approved for listing in China. We aimed to provide scientific evidence for policymakers to determine the most cost-effective vaccination strategy, to identify whether elderly individuals should be prioritized, to provide economic evidence for its scale-up in the Chinese setting, and to provide suggestions on the marketing and supply of vaccines.

## 2. Methods

### 2.1. Study Design

A decision-analytic Markov model using the susceptible–infectious–recovered (SIR) structure was developed to evaluate the cost-effectiveness of sequential vaccination compared with two-dose inactivated vaccination in China from a societal perspective. Base case and scenario analyses were performed to examine the situations stratified by virus strain and age, and sensitivity analyses were conducted to test the robustness of the model.

### 2.2. Target Population and Vaccination Strategies

**Figure 1:**
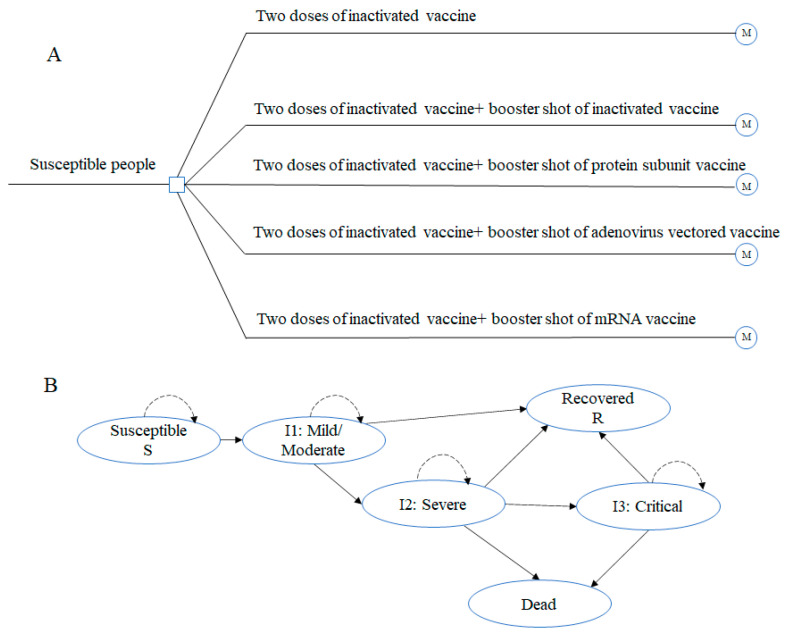
Decision-analytical Markov model using a susceptible–infectious–recovered (SIR) structure. (**A**) Decision-analytical Markov model; (**B**) Susceptible-Infected-Recovered (SIR) structure.

The target population of our model was adults aged 18 to 59 who had completed two-dose COVID-19 inactivated vaccination and were eligible for a sequential booster shot in China. We included four types of COVID-19 vaccines, and five vaccination strategies in total: the two-dose inactivated vaccine; booster vaccination with inactivated vaccine, adenovirus vectored vaccine, protein subunit vaccine, and mRNA vaccine (Figure 1A).

### 2.3. Model Structure and Assumptions

We constructed a model to stimulate the transition of the COVID-19 pandemic over a one-year time horizon, with a weekly cycle. Using a unidirectional SIR structure, six mutually exclusive health statuses were included (Figure 1B). We assumed that all target populations were susceptible to COVID-19 [30], so all participants were in the susceptible status when entering the model. Then, a proportion of them would become infected (I) if exposed to the virus. The infected state was further divided into three separate statuses: I_1_ denoted infection cases with mild or moderate symptoms; I_2_ denoted severe cases requiring hospitalization; and I_3_ denoted critical cases requiring intensive care unit (ICU) care [31]. Patients who survived infection went into the recovered state (R). Patients in I_2_ or I_3_ may die due to COVID-19 infection, and background mortality was not considered in the one-year time horizon. The model was developed using TreeAge Pro 2021.

The key assumptions of this study were as follows [32]: (1) the same distribution of COVID-19 outcomes across the target population; (2) no spontaneous elimination of the virus; (3) no influence of other nonpharmaceutical interventions (NPIs) on the transition of the disease; (4) all infected individuals would receive treatment; (5) individuals who recovered from COVID-19 infection would not relapse in the study period; (6) non-COVID-19 deaths were ignored during the study period; (7) asymptomatic individuals were included in the infected status and would be static until they recovered; and (8) the vaccination rate was 100% among different age groups in our base case and scenario analysis.

### 2.4. Model Parameters

#### 2.4.1. Vaccine Effectiveness

The effectiveness of two-dose inactivated vaccination was derived from a real-world study in Chile [33]. The effectiveness of a booster dose with an inactivated vaccine, adenovirus vectored vaccine, and mRNA vaccine came from an observational study in Chile [34]. Since there were no VE data of a booster dose with protein subunit vaccine, we calculated it based on the VE of full inoculation of protein subunit vaccine [35] and mRNA vaccine [36].

#### 2.4.2. Transition Probabilities

The natural infection rate of unvaccinated people was taken from a Chile real-world study (Table 1) [33]. The one-year probability of infection was converted by the incidence in the cohort by $p=1-e^{-r}$, where *p* is the one-year infection probability and *r* is the incidence rate (person-year) [8]. The transition probabilities between I_1_, I_2_, and I_3_ were obtained from the patient proportion in China [37]. The probability of recovery from different infection statuses came from a study using surveillance data of Chinese and American COVID-19 cases of the original strain [3].

#### 2.4.3. Costs

The societal perspective was adopted with both direct and indirect costs being included in this study, and all costs were converted to US$ using the official exchange rates of 2021 (US$1 = ￥6.449) [38] (Table 1). Cost and QALYs were not discounted due to the one-year time horizon.

The direct medical costs consisted of vaccination costs and medical costs. For the vaccination costs, vaccine procurement, cold-chain transportation, refrigeration, and administration were included. The price of vaccines equaled the lowest global purchase price published by the WHO [39] or news [40]. The transportation cost of the vaccine was assumed to be 6% of the purchase price [41]. The refrigeration cost of inactivated vaccine, adenovirus vectored vaccine, and protein subunit vaccine was calculated at US$0.18 [8], while the cost for the mRNA vaccine was higher at US$0.39 because it needs to be stored in the −70 °C incubator. The vaccine administration fee was US$1.55 per dose [42]. The medical costs covered diagnosis, treatment, hospitalization, and care expenditures, and increased as disease severity grew [43].

Indirect costs came from productivity losses and were calculated based on the average daily salary [44] and working time lost.

#### 2.4.4. Health Utilities

There was a lack of utility scores for different COVID-19 health statuses in the Chinese population. Thus, we used an Iran study focusing on the health utility value of patients with COVID-19, and the utility scores from the Iran population were more representative of the Asian population and covered all health statuses in our model [45]. The utility of people of susceptible statuses was referred to in the paper, focusing on the norms for EQ-5D-5L among the Chinese general population, given that the general population does not have full health [46]. In this paper, we only distinguished the health utilities from the status of disease progression but not from age and sex.

**Table 1:** Model parameters.

Parameter	Base Case Value	Lower Bound	Upper Bound	Distribution	Data Source
Vaccination effectiveness (%)					
Two doses of inactivated vaccine					
Against infection	65.90	65.20	66.60	Beta	Jara et al. 2021 [33]
Against hospitalization	87.50	86.70	88.20	Beta	
Against ICU	90.30	89.10	91.40	Beta	
Against death	86.30	84.50	87.90	Beta	
Two doses of inactivated vaccine + booster shot of inactivated vaccine					
Against infection	78.80	76.80	80.60	Beta	Jara et al. 2022 [34]
Against hospitalization	86.30	83.70	88.50	Beta	
Against ICU	92.20	88.70	94.60	Beta	
Against death	86.70	80.50	91.00	Beta	
Two doses of inactivated vaccine+ booster shot of protein subunit vaccine					
Against infection	83.22	74.90	91.54	Beta	Calculated
Against hospitalization	91.44	82.30	100	Beta	
Against ICU	91.63	82.47	100	Beta	
Against death	91.64	82.48	100	Beta	
Two doses of inactivated vaccine + booster shot of adenovirus vectored vaccine					
Against infection	93.20	92.90	93.60	Beta	Jara et al. 2022 [34]
Against hospitalization	97.70	97.30	98.00	Beta	
Against ICU	98.90	98.50	99.20	Beta	
Against death	98.10	97.30	98.60	Beta	
Two doses of inactivated vaccine+ booster shot of mRNA vaccine					
Against infection	96.50	96.20	96.70	Beta	Jara et al. 2022 [34]
Against hospitalization	96.10	95.30	96.90	Beta	
Against ICU	96.20	94.60	97.30	Beta	
Against death	96.80	93.90	98.30	Beta	
Transition probabilities without vaccination					
Natural infection rate	0.1043	0.0939	0.1147	Beta	Jara et al. 2021 [33]
I_1_ to I_2_	0.1450	0.1305	0.1595	Beta	Zhao et al. 2021 [37]
I_2_ to I_3_	0.2540	0.2286	0.2794	Beta	
I_2_ to death	0.0005	0.00045	0.00055	Beta	
I_3_ to death	0.0005	0.00045	0.00055	Beta	
I_1_ to recover	0.7475	0.6728	0.8223	Beta	Padula et al. 2021 [3]
I_2_ to recover	0.6500	0.5850	0.7150	Beta	
I_3_ to recover	0.5300	0.4770	0.5830	Beta	
Vaccination cost per dose (2021 USD)					
Inactivated vaccine	4.00	4.00	5.50	Gamma	WHO [39]; Calculated
Adenovirus vectored vaccine	15.00	9.63	15.00	Gamma	WHO [39]; Calculated
Protein subunit vaccine	19.54	2.12	19.54	Gamma	News [40]; Calculated
mRNA vaccine	6.75	3.80	6.75	Gamma	WHO [39]; Calculated
Cold-chain freight fee as a percentage of vaccine cost	6%	/	/	/	Chen et al. 2019 [41]
Refrigerator storage of 2–8 ℃ incubator	0.18	/	/	/	Jiang et al. 2022 [8]
Refrigerator storage of −70 ℃ incubator	0.39	/	/	/	Calculated
Administration	1.55	/	/	/	National medical insurance bureau [42]
Medical costs of health status (2021USD)					
I_1_	876.32	619.11	1804.12	Gamma	Jin et al. 2020 [43];Zhao et al. 2021 [37]
I_2_	8284.63	5852.95	17057.25	Gamma	
I_3_/Death	23469.03	16861.36	49139.19	Gamma	
Length of hospital stay (day)					
I_1_	14	/	/	/	Jin et al. 2020 [43];Zhao et al. 2021 [37]
I_2_	21	/	/	/	
I_3_	42	/	/	/	
Death	42	/	/	/	
Average salary per day (2021USD)	42.55	32.07	76.04	Gamma	National Bureau of Statistics [44]
Health utilities					
Susceptible	0.946	0.9461	1		Xie et al. 2022
I_1_	0.847	0.762	0.932	Beta	Alinia et al. 2021 [45]
I_2_	0.766	0.689	0.843	Beta	
I_3_	0.629	0.566	0.692	Beta	
Recover	0.896	0.806	0.986	Beta	
Death	0	/	/	/	

### 2.5. Model Analysis

#### 2.5.1. Base Case Analysis

The total costs and quality-adjusted life years (QALYs) were generated for each vaccination strategy, and then the incremental cost-effectiveness ratio (ICER) was calculated. It was compared with the willingness-to-pay (WTP) threshold of the gross domestic product (GDP) per capita of China in 2021 (US$ 12556.37) [44] to determine whether the booster vaccination strategy was cost-effective and which combination was the most cost-effective strategy. When there were dominant strategies, net monetary benefit (NMB) was evaluated instead.

Although WTP may vary with educational level, place of residence, and attitude towards disease, we still used a fixed threshold as commonly used in other studies [47]. In addition, we did not apply willingness-to-accept [48] in this study as we adopted a societal perspective, and no vaccination-related payments were required in the Chinese setting.

#### 2.5.2. Sensitivity Analysis

One-way sensitivity analysis and probabilistic sensitivity analysis (PSA) were performed to explore the robustness of the model. In the one-way sensitivity analysis, all cost parameters were varied by their 95% confidence interval, a specific range where available, or ±10% of the point estimated value. For the cost of vaccine per dose, we assumed the health insurance paid for the same price for a full vaccination procedure for different kinds of vaccines, and used this cost as the lower bound for different vaccines.

For PSA, Monte Carlo simulation (N = 1000 iterations) was used to assess the effects of changing multiple parameters simultaneously. Cost parameters were assumed to follow gamma distributions, and utilities, probabilities, and rates were assumed to follow beta distributions. The results were presented as cost-effectiveness acceptability curves to demonstrate the probability of being cost-effective for different vaccination strategies at various WTP thresholds.

#### 2.5.3. Scenario Analysis

Scenario analysis was performed to adjust the VE of vaccination under the Omicron strain pandemic and to examine the cost-effectiveness for elderly groups aged 60–69, 70–79, and over 80 years old. In the first scenario, Hong Kong [49,50] and Shanghai [51] real-world data under the Omicron strain pandemic were used to calculate the VE of sequential vaccination and the transition probabilities between each health status. The parameters for the Hong Kong situation are listed in Supplementary Materials Table S1, and those for the Shanghai situation are listed in Table S2. In the second scenario, the targeted population was adults aged over 60, and the VE among people aged over 60 and mortality of the elderly among different age groups (60–69, 70–79, 80+) were generated using Hong Kong real-world data [50,52] (Table S3).

## 3. Results

### 3.1. Base Case Analysis

Sequential vaccination after a two-dose inactivated COVID-19 vaccine with different types of booster doses generated more QALYs with lower costs and thus was cost-saving compared with the two-dose inactivated vaccine group. Moreover, the heterogeneous vaccination groups reduced the number of infection cases with lower costs compared with the homogeneous group. More precisely, compared with two-dose vaccination, a booster with an inactivated vaccine, protein subunit vaccine, adenovirus vectored vaccine, and mRNA vaccine increased 0.0075, 0.0110, 0.0208, and 0.0249 QALYs and saved US$163.96, US$261.73, US$583.21, and US$724.49, respectively (Table 2).

### 3.2. Sensitivity Analysis

**Figure 2:**
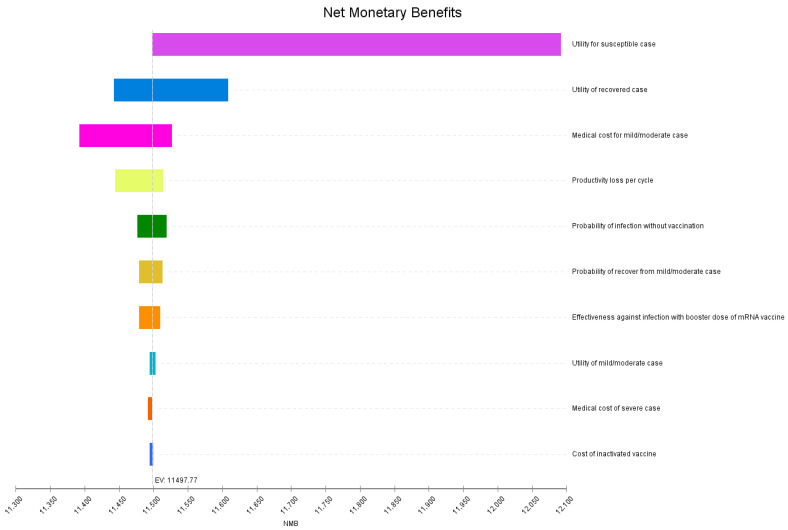
One-way sensitivity analyses of net monetary benefit for the model on willingness to pay.

The model results were robust, and the sequential vaccination strategy was always cost-saving under parameter variation. Cost parameters influenced the model more than effectiveness parameters (Figure S1). In addition, sensitivity analyses of NMB were generated to determine the top ten factors that influenced the model most, with utility for susceptible cases, the utility of recovered cases, and medical cost for mild/moderate cases ranked in the top three (Figure 2).

**Figure 3:**
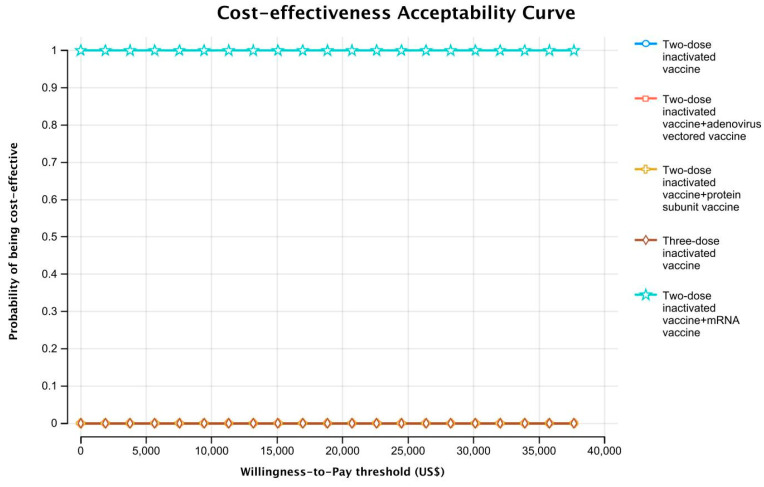
Cost-effectiveness acceptability curve.

In the PSA, since all sequential vaccination strategies were cost-saving compared with two-dose vaccination and a booster with mRNA vaccine can increase more QALYs at the lowest price, the probability for the sequential vaccination strategy using the mRNA vaccine as a booster shot being cost-effective was 100% compared with the other vaccination strategies (Figure 3).

### 3.3. Scenario Analysis

The results in scenario one showed that the total cost increased when the Omicron strain dominated the pandemic, and the booster vaccination strategy remained cost-saving. A booster of the mRNA vaccine could increase more QLAYs at the lowest price (Tables S4 and S5). Specifically, booster vaccination strategies in Hong Kong could save costs ranging from US$61.62 to US$99.95 and increase QALYs from 0.0046 to 0.0073. Meanwhile, for the situation in Shanghai, a booster vaccination could save costs ranging from US$14.83 to US$46.69 and increase QALYs from 0.0004 to 0.0011.

For scenario two, vaccination strategies remained effective and cost-saving in different age groups (60–69, 70–79, 80+) (Table S6). Labor loss due to illness was excluded from the model, and the total cost declined. The results indicated that increasing the coverage of booster vaccination among elderly people, especially octogenarians, dramatically decreased total costs. Hong Kong data illustrated that the percentage of severe disease and death increased dramatically with age among the elderly population without or with incomplete vaccination. Our results have shown that it is of great importance to provide sequential vaccination to elderly individuals, as the effectiveness of sequential vaccination strategies was proven to increase with age.

## 4. Discussion

This study developed an SIR status-adapted Markov model, and our analysis suggested that sequential vaccination was more effective at a lower cost than two-dose inactivated vaccination regardless of the type of booster dose. The findings are consistent with current prevention and control strategies in China and can provide crucial evidence to support decision-making.

The Markov model has been widely used to examine the cost-effectiveness of vaccination worldwide [3,4,52]. Although SIR or susceptible–exposed–infectious–recovered (SEIR) statuses were also adopted in their models, the effectiveness rate, the coverage rate, or the vaccine prices were assumed due to a lack of data. In addition, the assumption in our model that only patients with severe and critical diseases will die was much more reasonable than a previous study [4]. Our model included more comprehensive parameters and the latest data generated from real-world studies in the Chinese setting. To the best of our knowledge, this is the first cost-effectiveness analysis of COVID-19 sequential vaccination, and our results indicate that the sequential vaccination strategy is cost-saving compared with the two-dose vaccination.

By 7 September 2022, China reported 3433.96 million doses of COVID-19 vaccine, and 1270.66 million people have completed the initial inoculation, covering 90.13% of the total population; 8570.50 million people have received a booster shot, among which 45.72 million have competed for sequential vaccination [53]. Recently, the Omicron strain has become the most influential virus strain and has brought a huge burden to the world. The RWE from Hong Kong shows that people with incomplete vaccine inoculation or without vaccination accounted for 88.3% of total deaths, and most of the deceased cases were unvaccinated persons, especially the elderly [50]. Although VE against the Omicron strain was less effective than against other variants [18,25,54,55,56], RWE shows that homogeneous booster vaccination still has a very high-level protection rate, with VE against severe disease and death from approximately 92% [57] to 98% [49]. Although the pandemic patterns of Hong Kong and Shanghai are different, full inoculation and booster vaccination have been proven to be efficient in decreasing severe cases and deaths in a real-world setting, and our study can provide compelling cost-effectiveness evidence to support the sequential vaccination strategy, even when the Omicron strain dominated the pandemic.

Regarding the vaccination situation of the elderly in China, 227.44 million people aged over 60 years old completed the initial inoculation, reaching 86.14%, but only half of them received a booster dose of the COVID-19 vaccine [53]. They are more vulnerable but have relatively lower vaccination coverage, which may lead to an enormous risk of severe cases and deaths emerging under the Omicron pandemic. In this study, sequential vaccination strategies among different age groups were consistently effective and cost-saving, and adhering to and increasing the vaccination coverage rate with a booster dose should be encouraged to protect the elderly population. As this study would like to explore the cost-effectiveness of universal booster vaccination, the 100% coverage rate may slightly overestimate the effects of vaccination. The conclusion of this study was consistent with and supported by a recently published American study, which concluded that the booster vaccination strategy among elderly individuals was cost-saving compared to the two-dose mRNA vaccine without a booster [58].

Moreover, implementing a sequential vaccination strategy that gives priority to mRNA vaccines, adenovirus vectored vaccines and protein subunit vaccines and improves the vaccination coverage rate among elderly individuals, especially the rate of booster vaccination, is of great importance and is an effective way to prevent the outbreak of severe cases and deaths under the Omicron pandemic.

Our study has also shown that a booster shot of mRNA vaccines can increase more QALYs at the lowest price. Since mRNA vaccines are still unavailable in mainland China, the importation of mRNA vaccines or the marketing approval process of domestic mRNA vaccines should be accelerated. The price of mRNA used in our study can also be used as a reference for pricing.

The control of the Omicron strain pandemic has faced huge challenges. Further research on the exploration of the most cost-effective combination of vaccines and different NPI strategies in real-world scenarios, the reduction of unnecessary lockdown and containment policies, and the maximization of smooth society operation and economic development are needed to guarantee economic development, the sustainability of the health system, and to safeguard population well-being.

This study has some limitations. First, the medical costs of infected cases were obtained from the early stage of the COVID-19 outbreak in Wuhan, China. These data may change as the pandemic progresses, the diagnosis and treatment guidelines improve, and the infectivity and pathogenicity of the virus change. However, the Wuhan study contains the most complete and robust data on medical costs. Second, the transition probabilities between different health statuses were calculated using Hong Kong RWE; however, since mass testing of COVID-19 was not adopted there, the infection rate was underestimated, as asymptomatic cases could not be identified. Third, the SIR model cannot simulate the status from recovered to infected again, while in real-world settings, being recovered from COVID-19 can result in reinfection with another virus variant. A further limitation is that the simulation period was only one year, without considering the waning of effectiveness, since the adoption of the booster vaccination strategy lasted less than one year, and the evidence on the process and rates of the waning of effectiveness was lacking. Further analysis for a longer period considering the waning of vaccine effectiveness and mutating of virus strains is needed. Finally, due to the unavailability of the proportion of asymptomatic status, this study was not able to distinguish it from infected status.

## 5. Conclusions

The sequential vaccination strategy is cost-saving regardless of the type of vaccine in China as a real-world setting, and implementing the sequential vaccination of booster shots with heterogeneous vaccines can be given priority, in which a booster shot of mRNA vaccines is the most cost-saving strategy. Under the circumstances of the Omicron pandemic, improving the vaccination coverage rate among the elderly is of great importance in avoiding severe cases and deaths.

## 6. Patents

No patients or the public were involved in this study.

## Figures and Tables

**Table 2:** Cost-effectiveness analysis of different sequential vaccination strategies compared with two-dose inactivated vaccination.

Strategy	Cost (US$)	Effect (QALYs)	NMB (US$)	Incremental Cost (US$)	Incremental Effect (QALYs)	ICER (US$/QALY)
Two-dose inactivated vaccine	918.26	0.9062	9188.22	-	-	-
Two doses of inactivated vaccine+ booster shot of inactivated vaccine	755.30	0.9138	9435.36	−162.96	0.0075	−21,587.61
Two doses of inactivated vaccine+ booster shot of protein subunit vaccine	656.52	0.9172	9572.21	−261.73	0.0110	−23,875.83
Two doses of inactivated vaccine+ booster shot of adenovirus vectored vaccine	335.04	0.9271	10,003.44	−583.21	0.0208	−28,034.30
Two doses of inactivated vaccine+ booster shot of mRNA vaccine	193.77	0.9311	10,190.13	−724.49	0.0249	−29,123.71

## Data Availability

No new data were created or analyzed in this study. Data sharing is not applicable to this article.

## Supplementary Materials

The following supporting information can be downloaded at: https://www.mdpi.com/article/10.3390/vaccines10101712/s1, Figure S1: One-way sensitivity analyses for the model on ICER(US$/QALYs); Table S1: Model parameters under the Omicron strain pandemic; Table S2: Model parameters for people aged over 60 under the Omicron strain pandemic; Table S3: Cost-effectiveness analysis of different sequential vaccination strategies compared with initial inoculation under the Omicron strain pandemic; Table S4: Cost-effectiveness analysis of different sequential vaccination strategies among age-specific groups.

## References

[1] World Health Organization (WHO) Coronavirus COVID-19 Dashboard. https://covid19.who.int.

[2] Shaker M., Abrams E.M., Greenhawt M. (2021). A Cost-Effectiveness Evaluation of Hospitalizations, Fatalities, and Economic Out-comes Associated with Universal Versus Anaphylaxis Risk-Stratified COVID-19 Vaccination Strategies. J. Allergy Clin. Immunol. Pract..

[3] Padula W.V., Malaviya S., Reid N.M., Cohen B.G., Chingcuanco F., Ballreich J., Tierce J., Alexander G.C. (2021). Economic value of vaccines to address the COVID-19 pandemic: A U.S. cost-effectiveness and budget impact analysis. J. Med. Econ..

[4] Kohli M., Maschio M., Becker D., Weinstein M.C. (2021). The potential public health and economic value of a hypothetical COVID-19 vaccine in the United States: Use of cost-effectiveness modeling to inform vaccination prioritization. Vaccine.

[5] Marco-Franco J.E., Emilio J., Pita-Barros P., González-de-Julián S., Sabat I., Vivas-Consuelo D. (2021). Simplified Mathematical Modeling of Uncertainty: Cost-Effectiveness of COVID-19 Vaccines in Spain. Mathematics.

[6] Debrabant K., Grønbæk L., Kronborg C. (2021). The Cost-Effectiveness of a COVID-19 Vaccine in a Danish Context. Clin. Drug Investig..

[7] Hagens A., Inkaya A., Yildirak K., Sancar M., van der Schans J., Sancar A.A., Ünal S., Postma M., Yeğenoğlu S. (2021). COVID-19 Vaccination Scenarios: A Cost-Effectiveness Analysis for Turkey. Vaccines.

[8] Jiang Y., Cai D., Shi S. (2021). Economic evaluations of inactivated COVID-19 vaccines in six Western Pacific and South East Asian countries and regions: A modeling study. Infect. Dis. Model..

[9] Belayachi J., Obtel M., Razine R., Abouqal R. (2022). Long term effectiveness of inactivated vaccine BBIBP-CorV (Vero Cells) against COVID-19 associated severe and critical hospitalization in Morocco. medRxiv.

[10] Glöckner S., Hornung F., Baier M., Weis S., Pletz M.W., Deinhardt-Emmer S., Löffler B. (2021). CoNAN Study Group Robust Neutralizing Antibody Levels Detected after Either SARS-CoV-2 Vaccination or One Year after Infec-tion. Viruses.

[11] Levine-Tiefenbrun M., Yelin I., Alapi H., Katz R., Herzel E., Kuint J., Chodick G., Gazit S., Patalon T., Kishony R. (2021). Viral loads of Delta-variant SARS-CoV-2 breakthrough infections after vaccination and booster with BNT162b2. Nat. Med..

[12] Zeng G., Wu Q., Pan H., Li M., Yang J., Wang L., Wu Z., Jiang D., Deng X., Chu K. (2021). Immunogenicity and safety of a third dose of CoronaVac, and immune persistence of a two-dose schedule, in healthy adults: Interim results from two single-centre, double-blind, randomised, placebo-controlled phase 2 clinical trials. Lancet Infect. Dis..

[13] Abbasi J. (2022). Studies Suggest COVID-19 Vaccine Boosters Save Lives. JAMA.

[14] Guo W., Duan K., Zhang Y., Yuan Z., Zhang Y., Wang Z., Zhao D., Zhang H., Xie Z., Li X. (2021). Safety and immunogenicity of an inactivated SARS-CoV-2 vaccine in healthy adults aged 18 years or older: A ran-domized, double-blind, placebo-controlled, phase 1/2 trial. eClinicalMedicine.

[15] Pan H., Wu Q., Zeng G., Yang J., Jiang D., Deng X., Chu K., Zheng W., Zhu F., Yu H. (2021). Immunogenicity and safety of a third dose, and immune persistence of CoronaVac vaccine in healthy adults aged 18-59 years: Interim results from a double-blind, randomized, placebo-controlled phase 2 clinical trial. medRxiv.

[16] Falsey A.R., Frenck R.W., Walsh E.E., Kitchin N., Absalon J., Gurtman A., Lockhart S., Bailey R., Swanson K.A., Xu X. (2021). SARS-CoV-2 Neutralization with BNT162b2 Vaccine Dose 3. N. Engl. J. Med..

[17] Saban M., Myers V., Wilf-Miron R. (2022). Changes in infectivity, severity and vaccine effectiveness against delta COVID-19 vari-ant ten months into the vaccination program: The Israeli case. Prev. Med..

[18] Bar-On Y.M., Goldberg Y., Mandel M., Bodenheimer O., Freedman L., Kalkstein N., Mizrahi B., Alroy-Preis S., Ash N., Milo R. (2021). Protection of BNT162b2 Vaccine Booster against Covid-19 in Israel. N. Engl. J. Med..

[19] Barda N., Dagan N., Cohen C., Hernán M.A., Lipsitch M., Kohane I.S., Reis B.Y., Balicer R.D. (2021). Effectiveness of a third dose of the BNT162b2 mRNA COVID-19 vaccine for preventing severe outcomes in Israel: An observational study. Lancet.

[20] Sapkota B., Saud B., Shrestha R., Al-Fahad D., Sah R., Shrestha S., Rodriguez-Morales A.J. (2021). Heterologous prime–boost strategies for COVID-19 vaccines. J. Travel Med..

[21] Petrelli F., Luciani A., Borgonovo K., Ghilardi M., Parati M.C., Petrò D., Lonati V., Pesenti A., Cabiddu M. (2022). Third dose of SARS-CoV-2 vaccine: A systematic review of 30 published studies. J. Med. Virol..

[22] Thompson M.G. (2022). Effectiveness of a Third Dose of mRNA Vaccines Against COVID-19-Associated Emergency Department and Urgent Care Encounters and Hospitalizations Among Adults During Periods of Delta and Omicron Variant Predominance-VI-SION Network, 10 States, August 2021-January 2022. MMWR Morb. Mortal. Wkly. Rep..

[23] Hillus D., Schwarz T., Tober-Lau P., Vanshylla K., Hastor H., Thibeault C., Jentzsch S., Helbig E.T., Lippert L.J., Tscheak P. (2021). Safety, reactogenicity, and immunogenicity of homologous and heterologous prime-boost immunisation with ChA-dOx1 nCoV-19 and BNT162b2: A prospective cohort study. Lancet Respir. Med..

[24] Reimann P., Ulmer H., Mutschlechner B., Benda M., Severgnini L., Volgger A., Lang T., Atzl M., Huynh M., Gasser K. (2022). Efficacy and safety of heterologous booster vaccination with Ad26.COV2.S after BNT162b2 mRNA COVID-19 vaccine in haemato-oncological patients with no antibody response. Br. J. Haematol..

[25] Zuo F., Abolhassani H., Du L., Piralla A., Bertoglio F., de Campos-Mata L., Wan H., Schubert M., Wang Y., Sun R. (2022). Heterologous immunization with inactivated vaccine followed by mRNA booster elicits strong humoral and cellular immune responses against the SARS-CoV-2 Omicron variant. medRxiv.

[26] Kanokudom S., Assawakosri S., Suntronwong N., Auphimai C., Nilyanimit P., Vichaiwattana P., Thongmee T., Yorsaeng R., Srimuan D., Thatsanatorn T. (2022). Safety and Immunogenicity of the Third Booster Dose with Inactivated, Viral Vector, and mRNA COVID-19 Vaccines in Fully Immunized Healthy Adults with Inactivated Vaccine. Vaccines.

[27] Chiu N.-C., Chi H., Tu Y.-K., Huang Y.-N., Tai Y.-L., Weng S.-L., Chang L., Huang D.T.-N., Huang F.-Y., Lin C.-Y. (2021). To mix or not to mix? A rapid systematic review of heterologous prime–boost covid-19 vaccination. Expert Rev. Vaccines.

[28] Zhao Z., Cui T., Huang M., Liu S., Su X., Li G., Song T., Li W., Zhong N., Xu M. (2022). Heterologous boosting with third dose of coronavirus disease recombinant subunit vaccine increases neutralizing antibodies and T cell immunity against different severe acute respiratory syndrome coronavirus 2 variants. Emerg. Microbes Infect..

[29] Kaku C.I., Champney E.R., Normark J., Garcia M., Johnson C.E., Ahlm C., Christ W., Sakharkar M., Ackerman M.E., Klingström J. (2022). Broad anti-SARS-CoV-2 antibody immunity induced by heterologous ChAdOx1/mRNA-1273 vaccination. Science.

[30] Commission N.H. COVID-19 Diagnosis and Treatment Guideline (Version 9). http://www.nhc.gov.cn/yzygj/s7653p/202203/b74ade1ba4494583805a3d2e40093d88.shtml.

[31] China M.o.T.o.t.P.s.R.o. COVID-19 Prevention and Control Guidelines for Ports and Their Frontline Personnel (Ninth Edition). https://xxgk.mot.gov.cn/2020/jigou/syj/202203/t20220303_3644120.html.

[32] Zhou D., Shao T., Shao H., Tu Y., Tang Y., Zhou J., Malone D., Tang W. (2022). EPH172 When Is It Valuable for COVID-19 Booster Dose?: A Transmission Dynamics Model-Based Effectiveness and Cost-Effectiveness Analysis of Two Booster Dose Vaccination Priority Strategies in Mainland China. Value Health.

[33] Jara A., Undurraga E.A., González C., Paredes F., Fontecilla T., Jara G., Araos R. (2021). Effectiveness of an Inactivated SARS-CoV-2 Vaccine in Chile. N. Engl. J. Med..

[34] Jara A., Undurraga E.A., Zubizarreta J.R., González C., Pizarro A., Acevedo J., Araos R. (2022). Effectiveness of homologous and heterologous booster doses for an inactivated SARS-CoV-2 vaccine: A large-scale pro-spective cohort study. Lancet Glob. Health.

[35] Zhifei Biological Products Co. L. The Coronavirus Recombinant Protein Vaccine (CHO Cells) has been Approved for Conditional Marketing. http://www.zhifeishengwu.com/news/gsyw/qyyw/2022-03-02/624.html.

[36] Zheng C., Shao W., Chen X., Zhang B., Wang G., Zhang W. (2021). Real-world effectiveness of COVID-19 vaccines: A literature review and meta-analysis. Int. J. Infect. Dis..

[37] Zhao J., Jin H., Li X., Jia J., Zhang C., Zhao H., Ma W., Wang Z., He Y., Lee J. (2021). Disease Burden Attributable to the First Wave of COVID-19 in China and the Effect of Timing on the Cost-Effectiveness of Movement Restriction Policies. Value Health.

[38] Data O. (2022). Exchange Rates. https://data.oecd.org/conversion/exchange-rates.htm.

[39] World Health Organization Vaccine Purchase Data. https://www.who.int/teams/immunization-vaccines-and-biologicals/vaccine-access/mi4a/mi4a-vaccine-purchase-data.

[40] Finance S. In-depth Analysis of the Pharmaceutical Industry. http://stock.finance.sina.com.cn/stock/go.php/vReport_Show/kind/lastest/rptid/660793064184/index.phtml.

[41] Chen C., Liceras F.C., Flasche S., Sidharta S., Yoong J., Sundaram N., Jit M. (2019). Effect and cost-effectiveness of pneumococcal conjugate vaccination: A global modelling analysis. Lancet Glob. Health.

[42] Bureau N.M.I. Notice on the Effective Implementation of Phased Liquidation of Coronavirus Vaccines and Vaccination Costs. http://www.nhsa.gov.cn/art/2021/8/23/art_53_5856.html.

[43] Jin H., Wang H., Li X., Zheng W., Ye S., Zhang S., Zhou J., Pennington M. (2021). Economic burden of COVID-19, China, January-March, 2020: A cost-of-illness study. Bull. World Health Organ..

[44] Statistics N.B.o. China Statistical Data. https://data.stats.gov.cn/easyquery.htm?cn=C01.

[45] Alinia C., Yaghmaei S., Abdullah F.Z., Ahmadi A., Samadi N., Pourteimour S., Safari H., Mahmoodi H., Moradi G., Piroozi B. (2021). The health-related quality of life in Iranian patients with COVID-19. BMC Infect. Dis..

[46] Xie S., Wu J., Xie F. (2022). Population Norms for SF-6Dv2 and EQ-5D-5L in China. Appl. Health Econ. Health Policy.

[47] Harapan H., Anwar S., Bustamam A., Radiansyah A., Angraini P., Fasli R., Salwiyadi S., Bastian R.A., Oktiviyari A., Akmal I. (2017). Willingness to pay for a dengue vaccine and its associated determinants in Indonesia: A community-based, cross-sectional survey in Aceh. Acta Trop..

[48] Kabir K.M.A., Hagishima A., Tanimoto J. (2021). Hypothetical assessment of efficiency, willingness-to-accept and willing-ness-to-pay for dengue vaccine and treatment: A contingent valuation survey in Bangladesh. Hum. Vaccin. Immunother..

[49] Kong T.U.o.H. COVID-19 Vaccine Effectiveness in Hong Kong. http://www.med.hku.hk/en/news/press/-/media/D9C071B122C54C3089C5319E43E5187C.ashx.

[50] Hong Kong Special Administrative Region Government Archive of Statistics on 5th Wave of COVID-19. https://www.coronavirus.gov.hk/eng/5th-wave-statistics.html.

[51] Department of Health, The Government of the Hong Kong Special Administrative Region Health Fact of Hong Kong in 2021. https://www.dh.gov.hk/chs/statistics/statistics_hs/statistics_hfhk.html.

[52] Wang W.-C., Fann J.C.-Y., Chang R.-E., Jeng Y.-C., Hsu C.-Y., Chen H.-H., Liu J.-T., Yen A.M.-F. (2021). Economic evaluation for mass vaccination against COVID-19. J. Formos. Med. Assoc..

[53] Council S. Vaccination Status. http://www.gov.cn/xinwen/gwylflkjz190/index.htm.

[54] Yu X., Wei D., Xu W., Li Y., Li X., Zhang X., Qu J., Yang Z., Chen E. (2021). Reduced sensitivity of SARS-CoV-2 Omicron variant to booster-enhanced neutralization. medRxiv.

[55] Angkasekwinai N., Niyomnaitham S., Sewatanon J., Phumiamorn S., Sukapirom K., Senawong S., Toh Z.Q., Umrod P., Somporn T., Chumpol S. (2022). The immunogenicity and reactogenicity of four COVID-19 booster vaccinations against SARS-CoV-2 variants of concerns (Delta, Beta, and Omicron) following CoronaVac or ChAdOx1 nCoV-19 primary series. medRxiv.

[56] Ai J., Zhang H., Zhang Y., Lin K., Zhang Y., Wu J., Wan Y., Huang Y., Song J., Fu Z. (2022). Omicron variant showed lower neutralizing sensitivity than other SARS-CoV-2 variants to immune sera elicited by vaccines after boost. Emerg Microbes Infect..

[57] Huang Z., Xu S., Liu J., Wu L., Qiu J., Wang N., Ren J., Li Z., Guo X., Tao F. (2022). Effectiveness of inactivated and Ad5-nCoV COVID-19 vaccines against SARS-CoV-2 Omicron BA. 2 variant in-fection, severe illness, and death. medRxiv.

[58] Li R., Liu H., Fairley C.K., Zou Z., Xie L., Li X., Shen M., Li Y., Zhang L. (2022). Cost-effectiveness analysis of BNT162b2 COVID-19 booster vaccination in the United States. Int. J. Infect. Dis..

