# Supplementary material for: Cost-Effectiveness of COVID-19 Sequential Vaccination Strategies in Inactivated Vaccinated Individuals in China
Source: Vaccines (Basel). 2022 Oct 14;10(10):1712. doi: 10.3390/vaccines10101712 (PMC9610874; doi:10.3390/vaccines10101712)
Supplement: Supplementary file 1 [file vaccines-10-01712-s001.zip › vaccines-1906710-supplementary.pdf]

Supplementary material

Supplemental Table S1 Model parameters under the Omicron strain pandemic of Hong Kong

Parameter	Base-case Value	Lower bound	Upper bound	Distribution	Data source
**Two doses of inactivated vaccine (%)**					
Against infection	17.9	0	42.9	Beta	Hong Kong University[1]
Against hospitalization	91.7	87.8	94.4	Beta	
Against ICU	91.7	87.8	94.4	Beta	
Against death	94	89.6	96.5	Beta	
**Two doses of inactivated vaccine + booster shot of inactivated vaccine (%)**					
Against infection	42.3	11.4	62.4	Beta	Hong Kong University[1]
Against hospitalization	98.5	95.2	99.5	Beta	
Against ICU	98.5	95.2	99.5	Beta	
Against death	98.3	96.4	99.2	Beta	
**Two doses of inactivated vaccine+ booster shot of protein subunit vaccine (%)**					
Against infection	44.7	11.1	70.9	Beta	Calculated
Against hospitalization	100.0	93.6	100.0	Beta	
Against ICU	97.9	88.5	100.0	Beta	
Against death	100.0	98.8	100.0	Beta	
**Two doses of inactivated vaccine + booster shot of adenovirus vectored vaccine (%)**					
Against infection	50.0	13.8	72.5	Beta	Calculated
Against hospitalization	100.0	100.0	100.0	Beta	
Against ICU	100.0	100.0	100.0	Beta	
Against death	100.0	100.0	100.0	Beta	
**Two doses of inactivated vaccine+ booster shot of mRNA vaccine (%)**					
Against infection	51.8	14.3	74.9	Beta	Calculated
Against hospitalization	100.0	100.0	100.0	Beta	
Against ICU	100.0	100.0	100.0	Beta	
Against death	100.0	100.0	100.0	Beta	
**Transition probabilities without vaccination**					
Natural infection rate	0.1001			Beta	Government of Hong Kong[2] ; Department of Health[3]
I_1 to I_2	0.1014			Beta	
I_2 to I_3	0.3333			Beta	
I_2 to death	0.0031			Beta	
I_3 to death	0.0031			Beta	

Supplemental Table S2 Model parameters under the Omicron strain pandemic of Shanghai

Parameter	Base-case Value	Lower bound	Upper bound	Distribution	Data source
**Two doses of inactivated vaccine (%)**					
Against infection	16.3	15.4	17.2	Beta	Huang, et al, 2022[4]
Against hospitalization	88.6	85.8	90.9	Beta	
Against ICU	88.6	85.8	90.9	Beta	
Against death	91.7	86.9	94.7	Beta	
**Two doses of inactivated vaccine + booster shot of inactivated vaccine (%)**					
Against infection	19.2	18.2	20.3	Beta	Huang, et al, 2022[4]
Against hospitalization	92.7	90.1	94.6	Beta	
Against ICU	92.7	90.1	94.6	Beta	
Against death	95.9	91.4	98.1	Beta	
**Two doses of inactivated vaccine+ booster shot of protein subunit vaccine (%)**					
Against infection	20.3	17.7	23.1	Beta	Calculated
Against hospitalization	98.2	88.6	100.0	Beta	
Against ICU	92.1	83.8	100.0	Beta	
Against death	100.0	93.6	100.0	Beta	
**Two doses of inactivated vaccine + booster shot of adenovirus vectored vaccine (%)**					
Against infection	22.7	22.0	23.6	Beta	Calculated
Against hospitalization	100.0	100.0	100.0	Beta	
Against ICU	99.4	100.0	99.2	Beta	
Against death	100.0	100.0	100.0	Beta	
**Two doses of inactivated vaccine+ booster shot of mRNA vaccine (%)**					
Against infection	23.5	22.8	24.4	Beta	Calculated
Against hospitalization	100.0	100.0	100.0	Beta	
Against ICU	96.7	96.1	97.3	Beta	
Against death	100.0	100.0	100.0	Beta	
**Transition probabilities without vaccination**					
Natural infection rate	0.1001			Beta	Government of Hong Kong[2] ; Department of Health[3] ;
I_1 to I_2	0.0860			Beta	Huang, et al, 2022[4]; Shanghai Municipal Health Commission [5]
I_2 to I_3	0.0251			Beta	
I_2 to death	0.0098			Beta	
I_3 to death	0.0098			Beta	

Supplemental Table S3 Model parameters for people aged over 60 under the Omicron strain pandemic

Parameter	Infection	Hospitalization	ICU admission	Death	Data source
**Vaccination effectiveness (%)**					
Two doses of inactivated vaccine	17.9	72.2	72.2	77.4	Hong Kong University[1]
Two doses of inactivated vaccine + booster shot of inactivated vaccine	50.7	97.9	97.9	98.3	Hong Kong University[1]
Two doses of inactivated vaccine+ booster shot of protein subunit vaccine	53.5	100.0	97.3	100.0	Calculated
Two doses of inactivated vaccine + booster shot of adenovirus vectored vaccine	60.0	100.0	100.0	100.0	Calculated
Two doses of inactivated vaccine+ booster shot of mRNA vaccine (%)	62.1	100.0	100.0	100.0	Calculated
**Age specific infection and mortality among elderly (%)**					
60-69	15.79	0.23	0.31	0.04	Government of Hong Kong[2]；Department of Health[3]
70-79	14.60	0.37	0.74	0.17	
80+	15.42	0.68	1.76	1.18	

Supplemental Table S4 Cost-effectiveness analysis of different sequential vaccination strategies compared with initial inoculation under the Omicron strain pandemic of Hong Kong

Strategy	Cost (US$)	Effect (QALYs)	Incremental Cost (US$)	Incremental Effect (QALYs)	ICER (US$/QALY)
Two-dose inactivated vaccine	1027.18	0.8942	-	-	-
Two doses of inactivated vaccine+ booster shot of inactivated vaccine	962.85	0.8988	-64.33	0.0046	-14027.27
Two doses of inactivated vaccine+ booster shot of protein subunit vaccine	965.56	0.8994	-61.62	0.0052	-11843.71
Two doses of inactivated vaccine+ booster shot of adenovirus vectored vaccine	943.01	0.9009	-84.17	0.0067	-12559.44
Two doses of inactivated vaccine+ booster shot of mRNA vaccine	927.23	0.9014	-99.95	0.0073	-13765.14

Supplemental Table S5 Cost-effectiveness analysis of different sequential vaccination strategies compared with initial inoculation under the Omicron strain pandemic of Shanghai

Strategy	Cost (US$)	Effect (QALYs)	Incremental Cost (US$)	Incremental Effect (QALYs)	ICER (US$/QALY)
Two-dose inactivated vaccine	1035.67	0.8940	-	-	-
Two doses of inactivated vaccine+ booster shot of inactivated vaccine	1020.84	0.8944	-14.83	0.0004	-35376.66
Two doses of inactivated vaccine+ booster shot of protein subunit vaccine	1012.63	0.8946	-23.04	0.0006	-38820.21
Two doses of inactivated vaccine+ booster shot of adenovirus vectored vaccine	998.24	0.8949	-37.44	0.0010	-39000.53
Two doses of inactivated vaccine+ booster shot of mRNA vaccine	988.98	0.8950	-46.69	0.0011	-43027.77

Supplemental Table S6 Cost-effectiveness analysis of different sequential vaccination strategies among age-specific groups

Strategy	Cost (US$)	Effect (QALYs)	Incremental Cost (US$)	Incremental Effect (QALYs)	ICER (US$/QALY)
**Among elderly aged 60-69 years old**					
Two-dose inactivated vaccine	628.27	0.8847	-	-	-
Two doses of inactivated vaccine+ booster shot of inactivated vaccine	606.08	0.8864	-22.20	0.0017	-13065.30
Two doses of inactivated vaccine+ booster shot of protein subunit vaccine	620.55	0.8866	-7.73	0.0020	-3958.03
Two doses of inactivated vaccine+ booster shot of adenovirus vectored vaccine	615.73	0.8873	-12.55	0.0027	-4688.88
Two doses of inactivated vaccine+ booster shot of mRNA vaccine	607.19	0.8876	-21.09	0.0030	-7117.29
Among elderly aged 70-79 years old					
Two-dose inactivated vaccine	676.66	0.8854	-	-	-
Two doses of inactivated vaccine+ booster shot of inactivated vaccine	609.11	0.8877	-67.55	0.0023	-29252.10
Two doses of inactivated vaccine+ booster shot of protein subunit vaccine	620.54	0.8880	-56.13	0.0026	-21187.58
Two doses of inactivated vaccine+ booster shot of adenovirus vectored vaccine	615.68	0.8890	-60.98	0.0036	-16878.45
Two doses of inactivated vaccine+ booster shot of mRNA vaccine	607.11	0.8894	-69.55	0.0040	-17410.02
Among elderly aged over 80 years old					
Two-dose inactivated vaccine	842.79	0.8848	-	-	-
Two doses of inactivated vaccine+ booster shot of inactivated vaccine	615.24	0.8875	-227.55	0.0027	-85660.54
Two doses of inactivated vaccine+ booster shot of protein subunit vaccine	620.54	0.8878	-222.25	0.0030	-74393.55
Two doses of inactivated vaccine+ booster shot of adenovirus vectored vaccine	615.70	0.8887	-227.09	0.0039	-58036.58
Two doses of inactivated vaccine+ booster shot of mRNA vaccine	607.14	0.8891	-235.65	0.0043	-55063.58

Supplemental Figure S1

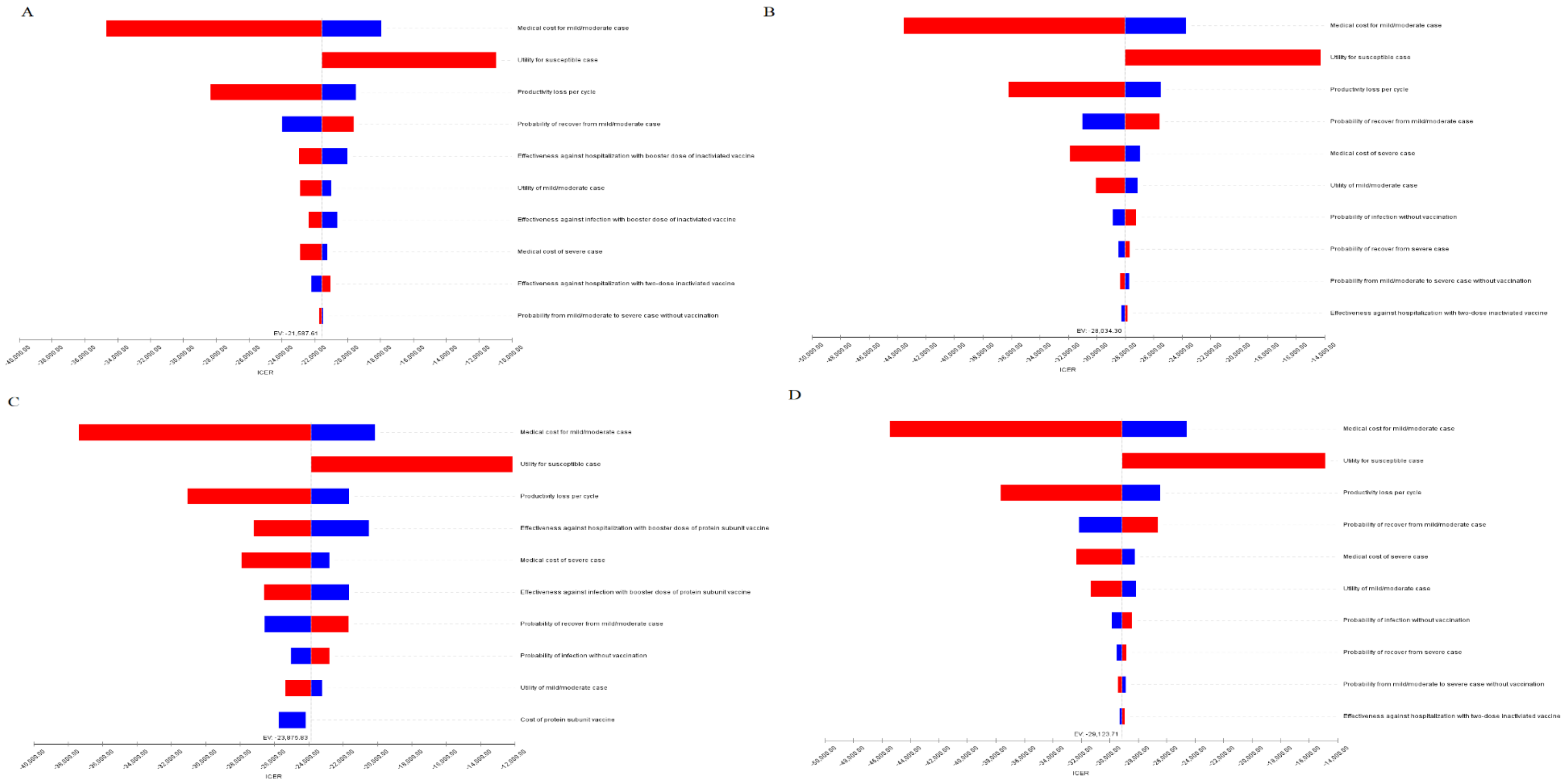

Figure S1 One-way sensitivity analyses for the model on ICER(US$/QALYs)

Figures A, B, C and D show the one-way sensitivity analyses of the model for sequential vaccination with a booster shot of inactivated vaccine, adenovirus vectored vaccine, protein subunit vaccine, and mRNA vaccine compared to two-dose inactivated vaccine.

1. The University of Hong Kong. *COVID-19 vaccine effectiveness in Hong Kong*. [cited 2022 April 5]; Available from: http://www.med.hku.hk/en/news/press/-/media/D9C071B122C54C3089C5319E43E5187C.ashx.
2. Hong Kong Special Administrative Region Government. *Archive of Statistics on 5th Wave of COVID-19*. [cited 2022 April 11]; Available from: https://www.coronavirus.gov.hk/eng/5th-wave-statistics.html.
3. The Government of the Hong Kong Special Administrative Region. *Health Fact of Hong Kong in 2021*. [cited 2022 April 12]; Available from: https://www.dh.gov.hk/chs/statistics/statistics_hs/statistics_hfhk.html.
4. Huang, Z., et al., https://www.dh.gov.hk/chs/statistics/statistics_hs/statistics_hfhk.html.*.* medRxiv, 2022: p. 2022.09.04.22279587.
5. Shanghai Municipal Health Commission. *The press release of COVID-19 pandemic*. [cited 2022 Sep 24]; Available from: https://wsjkw.sh.gov.cn/xwfb/index.html.
